# Early is Better: Report of a Cowden Syndrome

**DOI:** 10.1055/s-0043-1777275

**Published:** 2023-11-27

**Authors:** A. Di Nora, G. Pellino, A. Di Mari, F. Scarlata, F. Greco, P. Pavone

**Affiliations:** 1 Department of Clinical and Experimental Medicine, University of Catania, Catania, Italy; 2 Department of Biomedical and Biotechnological Sciences, Medical Genetics, University of Catania, Catania, Italy; 3 Department of Radiology, University of Catania, Catania, Italy

**Keywords:** overgrowth syndrome, PTEN gene, neuroimaging

## Abstract

In the clinical practice, it is not common for pediatricians to visit children with overgrowth phenotype. When it happens, it is important to focus on the age of manifestations and research the pathogenic causes using appropriate genetic test. Cowden syndrome is one of these rare causes; it is an autosomal dominant genodermatosis characterized by multiple hamartomas of ectodermal, mesodermal, and endodermal origin. It is caused by loss of function mutations in the phosphatase and tensin homolog (PTEN) gene located on chromosome 10q23.1 Loss of function of the PTEN gene contributes to overgrowth and risk for a variety of cancers including breast, thyroid, endometrium, skin, kidneys, and colon. The early diagnosis of Cowden disease allows a careful monitoring of the patients who are facing the risk of cancer transformation, which is the principal complication of the condition.

## Introduction

Cowden syndrome is an autosomal dominant genodermatosis described in 1963 by Lloyd and Dennis. It represents the most common phenotypical presentation of phosphatase and tensin homolog (PTEN) mutations. 1 The disease is characterized by multiple hamartomas, facial dysmorphism, mucocutaneous lesions, and macrocephaly.

These patients have an increased risk of developing malignant tumors, especially of the breast, thyroid, endometrium, kidney, and rectum. 2 3 4 The majority of mutations in this gene associated with PTEN hamartoma tumor syndrome are loss of function mutations. Many studies have also described the association of the PTEN gene with neurodevelopmental disorders and macrocephaly; in these last conditions there is often the presence of an altered but functional gene product. The protein plays an important role in regulating the duration of the cell cycle; in these patients, cell cycle appears shorter but with a higher proliferation rate, a lower response to stress with defects in migration, and differentiation of neuronal stem cells and neuronal precursor cell. 5 The protein intervenes in the processes of dendritic arborization, in the formation of synapses for neuronal circuits and in motor, memory, social interaction, and speech functions, which appear altered in these patients. 5

## Case Presentation, Genetic Analysis, and Neuroimaging

The patient, a 4-year-old girl, was evaluated for developmental delay. She was born at term after regular pregnancy with the weight of 3,600 g, head circumference 34 cm. Mother referred psychomotor development delay. At the clinical examination, we noted macrocrania associated with normal height and weight for growth curves (50° pc for age). As recommend in first line, we performed blood counts and complete biochemical analysis (IGF-I, IGFBP-3, free T4, and TSH) with normal result. For the suspect of a genetic syndrome because of the evidence of macrocrania and autistic disorders, we underwent genetic analysis of genes associated with overgrowth syndrome (next-generation sequencing gene panel). The analyses carried out the variant c.697c > T p.(Arg233*), a heterozygous mutation in the PTEN gene that results in a premature stop codon. The mutations was confirmed by Sanger sequencing. The variant is considered as “pathogenic” according to the American College of Medical Genetics and it is described as a pathogenic variant in different database (dbSNP, Clinvar, HGMD, LOVD, Franklin, Alamut, Varsome).

PTEN gene is a tumor suppressor gene located at chromosome 10q23.31, encoding for a protein of 403-amino acid with lipid and protein phosphatase activities and predominantly it is located in the cytoplasm. PTEN is the main antagonist of the phosphatidylinositol 3-phosphate kinase (PI3K)/protein kinase B (AKT) pathway by hydrolyzing phosphatidylinositol 3,4,5-triphosphate (PIP3) to phosphatidylinositol-4,5-bisphosphate (PIP2) and through this activity PTEN plays an important role in cellular proliferation and differentiation, in lipid and glucose metabolism and in regulation of many cellular functions. 6

In the most differentiated and resting cells, the protein is also located in the nucleus where PTEN has a role in chromosomal stability, DNA repair, and cell cycle regulation and PTEN inactivation can lead to genomic instability, apoptosis, and failure to repair DNA damage. 7 Loss of PTEN function can increase AKT activation causing cell proliferation and survival, and it plays a role in tumor development and progression.

After decades of study on the PTEN gene, recent studies have identified a new isoform of 173 amino-terminal extra amino acids that regulate mitochondrial energy metabolism. Recognition of isoform of PTEN helps to understand the complexity of PTEN function and it will advance our understanding on the role of PTEN in pathological processes. 8 Taking into account the multiple molecular effects of this protein, it is easy to understand how the mutations of this gene can be correlated to the etiology of different pathological conditions such as metabolic disorders, inflammatory and neurodegenerative conditions, onset of neoplasms, and neurodevelopmental disorders.

Monoallelic mutations in PTEN gene are associated with a PTEN hamartoma tumor syndrome, including Cowden syndrome, and with a hereditary form of autism associated with macrocephaly. These conditions are autosomal dominant genetic disorders, and these disorders are compatible with phenotypic spectrum of our patient.

**Fig. 1:**
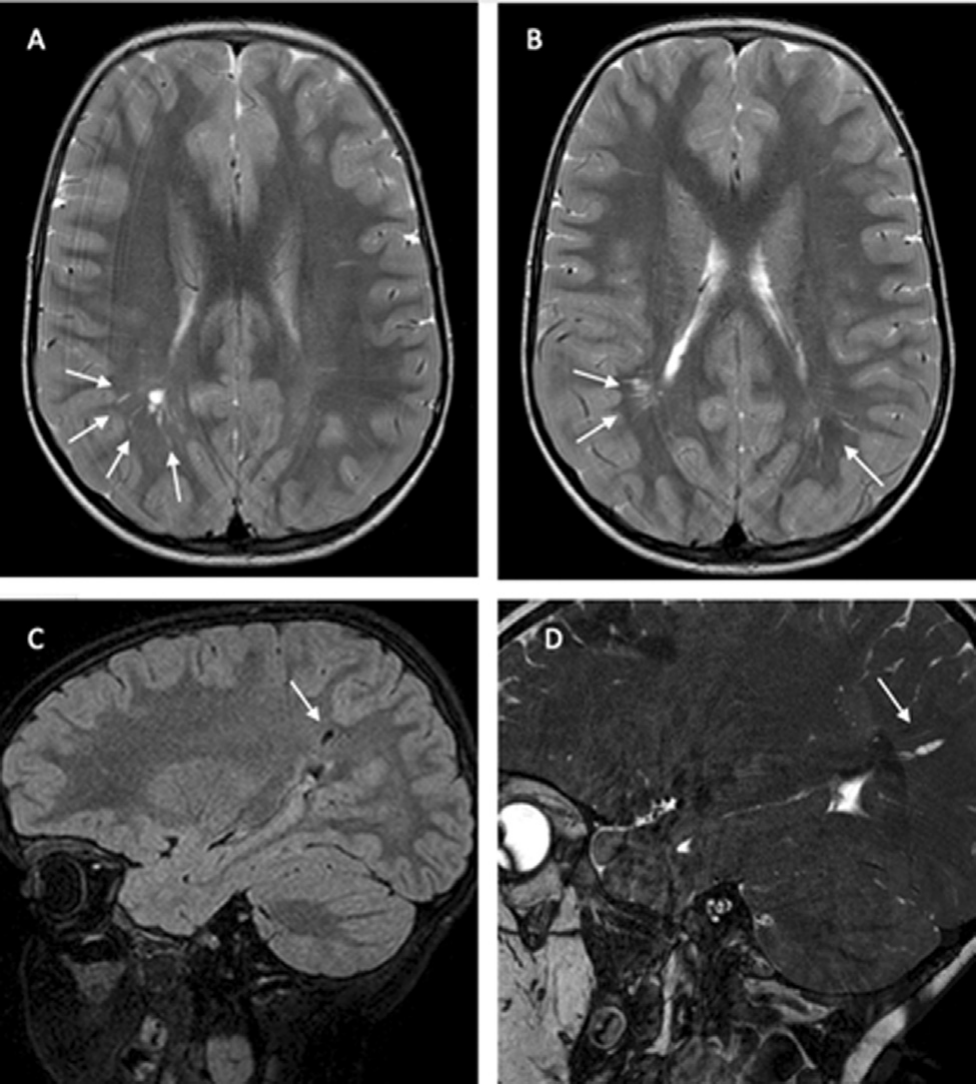
Axial T2-weighted TSE magnetic resonance imaging (MRI). ( **A–B** ) Sagittal fluid inversion recovery (FLAIR) MRI. ( **C–D** ) show bilateral posterior periventricular deep white matter linear region (prevailing in the right side) of high T2 signal with that attenuate fully on FLAIR due to prominent perivascular spaces (arrows).

We also performed a neuroimaging investigation ( Fig. 1 2 3 ), showing anomalies previous reported in literature. 9 In particular, we noted prominent perivascular spaces, white matter abnormalities, and frontal developmental venous anomalies in Cowden syndrome.

**Fig. 2:**
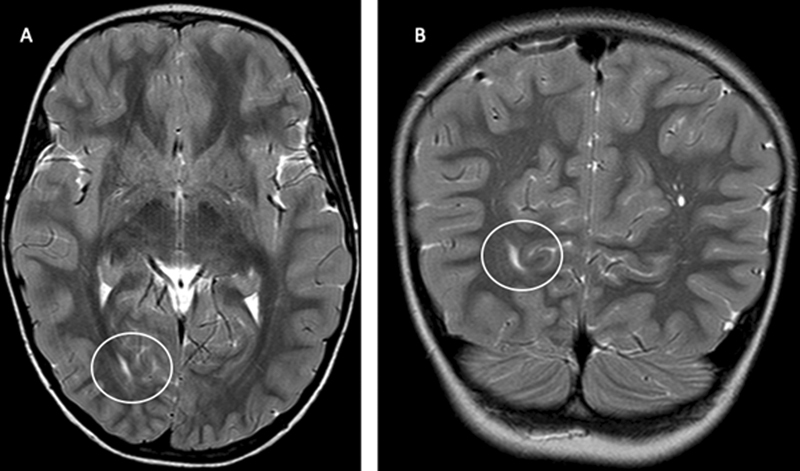
White matter abnormalities in Cowden syndrome. Axial ( **A** ) and coronal ( **B** ) T2-weighted magnetic resonance images show increased T2 signal intensity on right periventricular posterior region (circles).

**Fig. 3:**
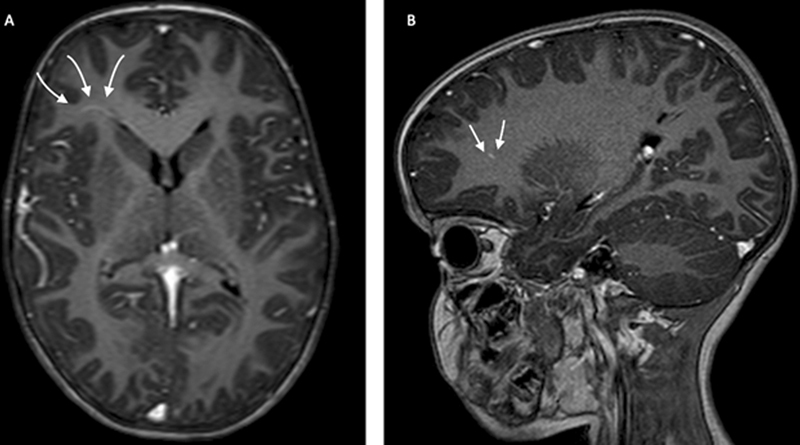
Frontal developmental venous anomalies in Cowden syndrome. Axial T1 ( **A** ) and sagittal T1 ( **B** ) postcontrast magnetic resonance imaging demonstrates right frontal developmental venous anomalies (arrows).

## Discussion and Conclusions

Genetic, epigenetic, and hormonal factors play a role in abnormally excessive growth. Overgrowth syndromes generally can present an increased risk of tumor predisposition that necessitate prompt diagnosis and appropriate referral. 10 For the clinical practice, we can summarize the overgrowth diagnosis into three phenotypes: prenatal, postnatal, and segmental overgrowth. 10

The prenatal overgrowth includes newborns who are large for gestational age. 11 Common considerations include maternal diabetes and overgrowth syndromes such as Beckwith–Wiedemann syndrome. Affected individuals may continue to show an accelerated growth postnatally (pre- and postnatal overgrowth) or may grow at a normal pace with length falling within 2 standard deviations (SDs) of the mean.

The postnatal overgrowth includes an accelerated growth pattern typically in childhood or adolescence. It usually depend on endogenous hormone-dependent growth (thyroid, growth hormone, sex hormones, or glucocorticoid). Other etiologies include familial tall stature (constitutional tall stature), precocious puberty, obesity, Marfan syndrome, homocystinuria, Klinefelter syndrome, and 47,XYY syndrome. 12

Finally, the segmental overgrowth is confined to one or a few regions of the body, such as macrocephaly in our case. Macrocephaly always requires special attention. PTEN syndrome disorders (Cowden syndrome, Bannayan–Riley–Ruvalcaba syndrome, and Proteus-like syndrome) are part of this group. As previous reported, PTEN is a phosphatase that removes a phosphate from the second messenger phosphatidylinositol triphosphate and, by doing so, inhibits the Akt (protein kinase B) pathway, a cardinal pathway of cell proliferation and angiogenesis. In literature, studies report different manifestation of segmental overgrowth syndrome. In particular, newborns with Bannayan–Riley–Ruvalcaba have striking macrocephaly (≥4.5 SD), out of proportion to their birth weight and length; those with Proteus-like presentation exhibit mosaic pattern of rapidly progressive overgrowth of different tissue types; Cowden syndrome, rarely expressed in children, associated with hamartomata and macrocephaly. 10 Typically, Cowden syndrome manifests mostly in second–third decade of life with distinctive trichilemmomas (benign neoplasm derived from the outer root sheath epithelium of the hair follicle), papillomatous papules (benign neoplasm of epithelium), and acral and plantar keratosis seen in 99% of patients. Thus, an early diagnosis such as our case is rarely reported, but it is important for the management including mucocutaneous manifestations and cancer surveillance.

As reported in literature, cerebral white matter anomalies are present in Cowden syndrome. 9 13 The mechanism behind enlarged perivascular spaces in Cowden syndrome is not clear, although they are seen in other genetic syndromes such as storage disorders (mucopolysaccharidosis). 9 PTEN has a role also in angiogenesis; thus, it frequently have vascular anomalies such as our patient.

In conclusion, Cowden syndrome is a rare presentation in pediatric age, but it should be suspected in case of macrocrania and developmental delay. A neuroimaging study is important, because of the risk of malignancy associated. An early diagnosis in pediatric age can modify the prognosis in adulthood.

## References

[1] Garofola C Jamal Z Gross G P Cowden disease Treasure Island (FL) StatPearls Publishing 2023. Accessed March 27, 2003 at:https://www.ncbi.nlm.nih.gov/books/NBK525984/

[2] Mahdi H Mester J L Nizialek E A Ngeow J Michener C Eng C Germline PTEN, SDHB-D, and KLLN alterations in endometrial cancer patients with Cowden and Cowden-like syndromes: an international, multicenter, prospective study Cancer 2015 121 05 688 696 25376524 10.1002/cncr.29106 PMC4339629

[3] Tan M H Mester J L Ngeow J Rybicki L A Orloff M S Eng C Lifetime cancer risks in individuals with germline PTEN mutations Clin Cancer Res 2012 18 02 400 407 22252256 10.1158/1078-0432.CCR-11-2283 PMC3261579

[4] Cavaillé M Ponelle-Chachuat F Uhrhammer N Early onset multiple primary tumors in atypical presentation of Cowden syndrome identified by whole-exome-sequencing Front Genet 2018 9 353 30233642 10.3389/fgene.2018.00353 PMC6127642

[5] Rademacher S Eickholt B J PTEN in autism and neurodevelopmental disorders Cold Spring Harb Perspect Med 2019 9 11 a036780 31427284 10.1101/cshperspect.a036780 PMC6824399

[6] Chen C Y Chen J He L Stiles B L PTEN: tumor suppressor and metabolic regulator Front Endocrinol (Lausanne) 2018 9 338 30038596 10.3389/fendo.2018.00338 PMC6046409

[7] Ming M He Y Y PTEN in DNA damage repair Cancer Lett 2012 319 02 125 129 22266095 10.1016/j.canlet.2012.01.003 PMC3326178

[8] Liang H He S Yang J PTENα, a PTEN isoform translated through alternative initiation, regulates mitochondrial function and energy metabolism Cell Metab 2014 19 05 836 848 24768297 10.1016/j.cmet.2014.03.023 PMC4097321

[9] Dhamija R Weindling S M Porter A B Hu L S Wood C P Hoxworth J M Neuroimaging abnormalities in patients with Cowden syndrome: retrospective single-center study Neurol Clin Pract 2018 8 03 207 213 30105160 10.1212/CPJ.0000000000000463 PMC6075984

[10] Manor J Lalani S R Overgrowth syndromes-evaluation, diagnosis, and management Front Pediatr 2020 8 574857Erratum in: Front Pediatr. 2020;8:624141 33194904 10.3389/fped.2020.574857 PMC7661798

[11] Yachelevich N Generalized overgrowth syndromes with prenatal onset Curr Probl Pediatr Adolesc Health Care 2015 45 04 97 111 25861999 10.1016/j.cppeds.2015.02.005

[12] Richmond E J Rogol A D The child with tall stature and/or abnormally rapid growth 2018Accessed April 2020 at:http://www.uptodate.com

[13] Dhamija R Hoxworth J M Imaging of PTEN-related abnormalities in the central nervous system Clin Imaging 2020 60 02 180 185 31927175 10.1016/j.clinimag.2019.12.006

